# Temporal trends in reptile occurrence among temperate old-growth, regrowth and replanted woodlands

**DOI:** 10.1371/journal.pone.0291641

**Published:** 2023-09-28

**Authors:** David B. Lindenmayer, Daniel Florance, David Smith, Clare Crane, Angelina Siegrist, Eleanor Lang, Mason Crane, Damian R. Michael, Ben C. Scheele, Maldwyn John Evans

**Affiliations:** 1 Sustainable Farms, Fenner School of Environment & Society, The Australian National University, Canberra, Australia; 2 NSW Biodiversity Conservation Trust, Gundagai, NSW, Australia; 3 Gulbali Institute for Agriculture, Water and Environment, Charles Sturt University, Albury, NSW, Australia; Feroze Gandhi Degree College, INDIA

## Abstract

Reptiles are an important part of the vertebrate fauna in the temperate woodlands of south-eastern Australia. However, compared to birds and mammals, the long-term occurrence of reptiles across woodland growth types–old growth, regrowth, and replantings–remains poorly understood. Here, using 18-years of data gathered at 218 sites across 1.5 million hectares in New South Wales South West Slopes bioregion, we sought to quantify patterns of temporal change in reptile occurrence and determine if such changes varied between woodland growth types. Despite extensive sampling, almost 75% of our 6341 surveys produced no detections of reptiles. Significant survey effort exceeding 2000 surveys was needed over a prolonged period of time to record detections of 26 reptile species in our study area. Our analyses showed a temporal increase in estimated reptile species richness and abundance over 18 years. Such increases characterized all three vegetation structural types we surveyed. At the individual species level, we had sufficient data to construct models for five of the 26 species recorded. Three of these species were least commonly detected in replantings, whereas the remaining two were most often detected in replantings relative to old growth and regrowth woodland. We found evidence of a temporal increase in two skink species, a decline in one gecko species, and no change in the remaining two skink species. Although detections were consistently low, active searches were the best survey method, and we suggest using this method in habitats known to be hotspots for reptiles, such as rocky outcrops, if the aim is to maximize the number of individuals and species detected. Our findings highlight the value of all three broad vegetation structure types in contributing to woodland reptile biodiversity.

## Introduction

Australia is a global hotspot for reptiles [1] as they are the most species-rich terrestrial vertebrate group in Australia with more than 1000 taxa described (www.arod.com.au/arod/reptilia/). Indeed, approximately 15 new species are added to the nations’ list of reptiles every year [2]. Despite the high level of reptile diversity in many Australian ecosystems [1, 3], there is a paucity of data on temporal change in detection and/or occurrence patterns in the majority of the nation’s environments [4–6], [but see 7, 8]. Yet, such data are critical for the conservation of those species undergoing declines and/or those taxa in need of targeted management action [9, 10].

Here, we report the results of long-term surveys of reptiles in the temperate woodlands of the South West Slopes (SWS) bioregion of south-eastern New South Wales, south-eastern Australia. These temperate woodlands are key habitats for a wide range of reptile species from many families [11–13]. However, the SWS bioregion is one of the most heavily modified large-scale biomes on Earth [14]. Extensive areas of woodland have been cleared or grazed [15], largely to create semi-cleared paddocks for domestic livestock grazing or cropping [16]. The impacts of such major changes in vegetation cover on populations of reptiles are poorly known, but are likely to be substantial and negative [12, 17], [although some studies have documented only muted effects of grazing on reptiles; e.g. 18, 19]. Negative impacts of land clearing would be expected given the loss of key components of habitat in temperate woodland environments such as large old trees [and associated bark habitats; see 20], and fallen timber [8, 21]. Threats from agriculture in general are estimated to affect over 12% of Australian reptiles [3] and more than 45% of species listed under IUCN threat categories [22]. Notably, in some parts of our study area on the SWS, there has been an increase in native woody vegetation cover over the past 3–4 decades, whereas in others it has declined [16, 23]. On this basis, the first key question with which we underpinned this study was: *What are the patterns of temporal change in species richness and the presence and abundance of reptiles in the temperate woodlands of the SWS bioregion*?

As a consequence of past land clearing, and efforts to mitigate clearing effects such as facilitating natural regeneration [24], and deliberate revegetation or replanting [25], there are three broad structural types of temperate woodland vegetation that characterise the SWS [26, 27]. These are old growth woodland, regrowth woodland (regenerated after past clearing), and replanted woodland that has developed from deliberate establishment of seedlings or seeding. There are marked differences in the structure of old growth, regrowth, and replanted woodlands [28], and whilst these differences can have major impacts on bird biota [26, 29] and some mammal fauna [30], how reptiles respond to these broad structural types remains poorly understood [13, 17, 31], [but see 32, 33]. Therefore, our second key question was: *Are there differences among the three broad types of vegetation types in reptile species richness and the presence and abundance of individual reptile taxa*?

Finally, there can be marked differences in the rate and direction of temporal change in the structure and composition of the three major types of vegetation cover in temperate woodlands [28]. This may, in turn, influence patterns of temporal suitability of different types of vegetation as habitat for biodiversity, as seen in earlier work for birds [see 26], but which also may influence other groups such as reptiles. Therefore, our third question was: *Are there broad vegetation type differences in temporal patterns of reptile species richness and the presence/abundance of individual reptile taxa*? Evidence of such effects would be reflected by an interaction between time and vegetation type and, for example, restoration efforts that lead to the establishment of vegetation structure such as replantings or natural regeneration may help speed the recovery process for reptiles [e.g. see 21].

Understanding where species occur and patterns of temporal change in species occurrence is important to guide efforts to conserve biodiversity and meet conservation objectives [4, 5, 10]. This includes in threatened ecological communities such as temperate woodlands where there are major challenges in vegetation restoration at the same time as meeting livestock production and cropping objectives as part of sustainable agricultural production [25].

## Methods

### Study area

**Fig 1:**
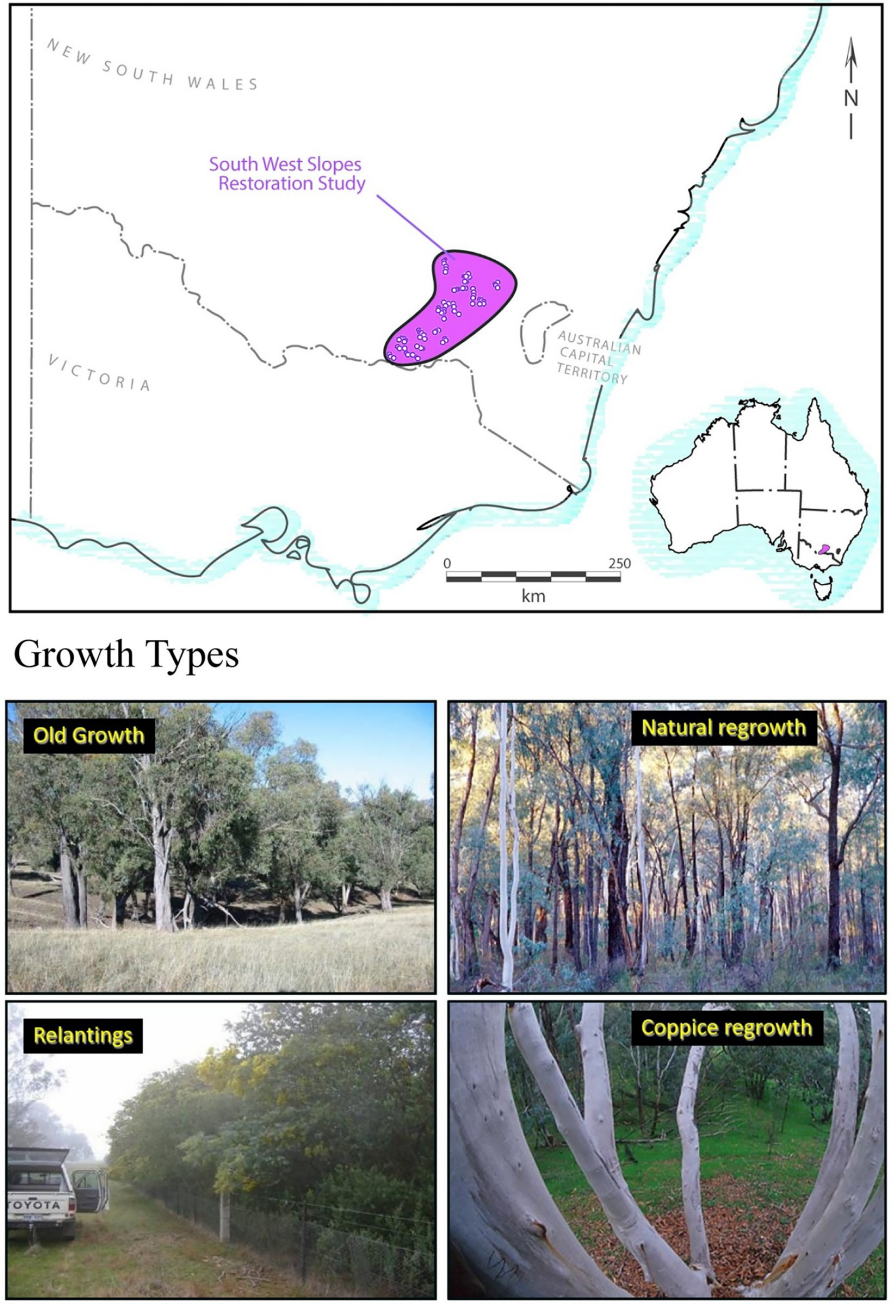
(Above) Location of the study area and long-term field sites in the South West Slopes (SWS) bioregion of south-eastern Australia. (Below) Photo images show the four key broad types of vegetation that were examined in this study.

Our study encompassed a 1.8 million ha agricultural area (spanning 150 km north-south and 120 km east-west) within the SWS bioregion of New South Wales, south-eastern Australia (Fig 1). The region was dominated by temperate woodland at the time of European colonization more than 220 years ago [34], but has been cleared of an estimated 85% of its original cover to facilitate livestock grazing and cereal cropping [15]. Remnant patches of temperate woodland vegetation are often highly disjunct and small (< 3 ha in size and typically far smaller) [23, 35]. Temperate woodland in south-eastern Australia (including in the SWS bioregion) is one of the most heavily modified biomes worldwide [14], and the agricultural regions where it used to be a predominant form of land cover are now characterised by a range of land degradation problems such as biodiversity loss, secondary salinity, soil erosion, and colonization of invasive species. In an effort to tackle the many land degradation problems in temperate woodlands, major restoration programs have been undertaken [36]. There also has been substantial natural regeneration of temperate woodlands, particularly over the past 15 years [24, 27], often as a result of changes in livestock grazing pressure [14]. Thus, woody vegetation in the SWS can be grouped into three broad categories: actively replanted woodland (restoration replantings), naturally regenerated or coppice woodland (regrowth woodland), and old-growth woodland [26] (Fig 1). We further describe below the ages and other attributes of the three broad vegetation types targeted for study in this investigation.

Our investigation was based on 218 long-term field sites, each of 1 ha in size and comprising a 200 m long and 50 m wide transect. The size of our sites was broadly matched to the typical size of woodland patches that characterize the heavily modified agricultural areas of south-eastern Australia [35]. Our 218 sites were located on 46 farms (Fig 1), and comprised 65 restoration replanting sites, 67 regrowth woodland sites, and 86 old-growth woodland sites. We located each site wholly within a given wooded area (i.e. within a replanting, area of regrowth woodland, or an area of old growth woodland).

Restoration replantings were areas of planted understory, midstory and overstory native vegetation with a mix of existing native and exotic ground cover. Woody plants were typically spaced 2–5 m apart, but there was not a standard set of spacing and plant species composition protocols applied in revegetation efforts. All restoration replantings were at least seven years old at the start of this investigation (2002) and many were 10–20 years old at that time. Most restoration replantings had been established to mitigate problems associated with soil erosion and/or secondary salinity.

Regrowth woodland refers to two kinds of natural regeneration. These are existing living trees recovering after disturbance by fire or partial clearing (termed coppice regrowth), or regeneration of trees in areas from seeds germinating after being dropped by overstory trees (termed seedling regrowth). These areas had largely been cleared except for the retention of scattered paddock trees. Areas of regrowth woodland were generally of intermediate age between replanted woodlands and old growth woodlands in our study. Although coppice regrowth and seedling regrowth woodland have different disturbance histories, we elected to combine them for subsequent statistical analyses (to ensure sufficient numbers of replicate sites in each woodland categories).

Old-growth woodland stands were typically dominated by large scattered trees that were 200 or more years old. Many of these areas have a heavily degraded ground layer with the shrub layer often missing as a result of intensive livestock grazing.

### Survey protocols

We employed two kinds of surveys for reptiles on our long-term sites–active searches and artificial substrate arrays. The artificial substrate arrays comprised two 1 m x 1 m pieces of corrugated iron, four standard concrete roof tiles, and four hardwood timber railway sleepers. We established an artificial substrate array at the 0 m and 100 m points along the 200 m transect at each of our 218 sites [37].

In each of our nine survey years between 2002 and 2020, we surveyed reptiles using repeated time and area-constrained (20 min/1 ha) active searches of natural habitat and inspections of two arrays of substrates. The time required to check each substrate array was approximately two and half minutes and hence five minutes per site [37].

Active searches included scanning a site for basking animals, raking through leaf litter, lifting logs and surface rocks, and inspecting exfoliating bark and rock crevices. For all survey years, up to four experienced observers visited eight sites each per day over a five-day period. All surveys were conducted on clear, generally sunny days between 0900 hours and 1400 hours [37].

### Animal ethics statement

The study was carried out in strict accordance with the recommendations made by the latest edition of the Australian Code of Practice for the Care and Use of Animals for Scientific Purposes, and as such, involves consideration of the steps taken by the applicant to comply with the principles of the 3Rs (Reduction, Replacement and Refinement). The animal ethics protocol was approved by the Australian National University Animal Experimentation Ethics Committee (Protocol Number: A2021/54).

### Covariates for use in statistical analyses

We constructed Bayesian generalized linear models of reptile abundance and the occurrence of individual reptile species with a particular focus on associations with the following potential explanatory variables: **(1)** the type of artificial substrate (timber, tiles, corrugated iron, pooling the samples from the 0 m and 100 m points) and active search, **(2)** growth type (old growth, regrowth, or replanting), and **(3)** time (survey year). We also sought to determine if the effect of year varied between growth types; that is, if there was evidence of a growth type x year interaction in which detections increased or decreased faster in a particular growth type relative to another growth type.

### Statistical analyses

We constructed Bayesian generalized linear mixed models using the ‘brms’ package [38] in R [39]. For overall reptile abundance and species richness, we fitted hurdle negative binomial mixed models. Hurdle negative binomial models consist of two components: **(1)** a probability of a zero component, which was modelled as a logistic regression, and **(2)** a conditional counts component, which, in this case, was modelled as a zero-truncated negative-binomial regression [40]. The model outputs three components of the estimates: the hurdle estimates (using presence/absence data), the conditional estimates (using zero-truncated data), and the unconditional estimates (all data).

We detected five species of reptiles sufficiently frequently (> 1% of surveys) to enable the construction of robust statistical models. These were four scincids: *Morethia boulengeri*, *Ctenotus spaldingi*, *Carlia tetradactyla*, and *Cryptoblepharus pannosus*, and one gecko, *Christinus marmoratus*.

To model reptile species responses to time, growth types, and sampling technique, we used binomial response data (i.e., presence/absence data—whether in any given survey the species is detected once or more). We fitted these models assuming a Bernoulli distribution.

For each response variable, we fitted a model to test the additive linear effects of growth type (*G*_*i*_), time (*T*_*t*_), and sampling technique (*S*_*i*_). Using the binomial component of the models as an example, we assumed that:

$$logit(\psi_{i,t})=\beta_{0}+\beta_{1}G_{i}+\beta_{2}T_{t}+\beta_{3}S_{i}+u_{i}+y_{i},$$

where *ψ*_*i*,*t*_ is the probability of occurrence at site *i*, and year *t*. *β*_0_ is the intercept and *β*_1_ to *β*_3_ are the associated regression coefficients representing the linear effects of the three predictor variables. We included site- and farm-level random effects which allowed for dependence between years (*u*_*i*_+*y*_*i*_). We fitted a second model to test for an interactive effect between time and growth type:

$$logit(\psi_{i,t})=\beta_{0}+\beta_{1}G_{i}+\beta_{2}T_{t}+\beta_{3}S_{i}+\beta_{4}T_{t}G_{i}+u_{i}+y_{i},$$

comparing this model’s fit to the earlier model using leave-one-out cross validation information criterion scores (LOOIC) [41]. We chose the most parsimonious model i.e., the simplest model within two LOOIC scores of the best-fitting model [41, 42]. We fitted all models with student-t priors specifying four chains, 2,000 iterations, including 500 warm-up/burn-in iterations.

### Species accumulation curves

We computed species accumulation curves separately for sites overall, within each growth type, and using different search techniques. To construct the curves, we used the ‘specaccum’ function in the ‘vegan’ package [43] in R [39].

## Results

Our surveys produced very low levels of detections; from 6341 surveys there were 4816 surveys (74.9%) with zero species recorded, 1128 surveys (17.8%) in which one species was detected, 304 surveys (4.8%) in which two species were detected, 73 surveys (1.2%) with three species detected, 15 surveys (0.2%) with four species detected, and five surveys (0.08%) where five species were recorded. Active searches yielded the highest detections (47.6%), whereas tiles were the least effective at detecting reptiles (13.7%) (Table 1).

**Table 1:** Summary of the number of detections of reptiles by sampling technique. Percentages in parentheses represent the percentage of searches with a detection of that species (Taxonomic nomenclature follows Wilson and Swan 2020 [44]).

Family	Species	Active Search	Tiles	Timbers	Tins
Agamidae	*Pogona barbata*	14 (0.88%)	0 (0.00%)	0 (0.00%)	1 (0.06%)
Carphodactylidae	*Underwoodisaurus milii*	3 (0.19%)	1 (0.06%)	1 (0.06%)	0 (0.00%)
Diplodactylidae	*Diplodactylus vittatus*	22 (1.38%)	17 (1.08%)	6 (0.38%)	6 (0.38%)
	*Strophurus intermedius*	0 (0.00%)	0 (0.00%)	0 (0.00%)	3 (0.19%)
Gekkonidae	*Christinus marmoratus*	171 (10.70%)	4 (0.25%)	9 (0.57%)	46 (2.91%)
Pygopodidae	*Delma inornata*	17 (1.06%)	17 (1.08%)	6 (0.38%)	15 (0.95%)
	*Aprasia parapulchella*	3 (0.19%)	1 (0.06%)	0 (0.00%)	0 (0.00%)
Scincidae	*Morethia boulengeri*	339 (21.21%)	120 (7.59%)	71 (4.49%)	254 (16.07%)
	*Cryptoblepharus pannosus*	281 (17.58%)	1 (0.06%)	9 (0.57%)	3 (0.19%)
	*Carlia tetradactyla*	110 (6.88%)	25 (1.58%)	9 (0.57%)	61 (3.86%)
	*Ctenotus spaldingi*	67 (4.19%)	12 (0.76%)	7 (0.44%)	14 (0.89%)
	*Hemiergis talbingoensis*	24 (1.50%)	17 (1.08%)	25 (1.58%)	22 (1.39%)
	*Egernia striolata*	54 (3.38%)	0 (0.00%)	0 (0.00%)	8 (0.51%)
	*Lerista timida*	8 (0.50%)	6 (0.38%)	0 (0.00%)	1 (0.06%)
	*Lerista bougainvillii*	5 (0.31%)	6 (0.38%)	2 (0.13%)	0 (0.00%)
	*Tiliqua scincoides* ssp. *scincoides*	3 (0.19%)	0 (0.00%)	2 (0.13%)	9 (0.57%)
	*Menetia greyii*	7 (0.44%)	1 (0.06%)	0 (0.00%)	2 (0.13%)
	*Lampropholis delicata*	4 (0.25%)	1 (0.06%)	0 (0.00%)	2 (0.13%)
	*Lampropholis guichenoti*	3 (0.19%)	0 (0.00%)	0 (0.00%)	1 (0.06%)
Varanidae	*Varanus varius*	13 (0.81%)	0 (0.00%)	0 (0.00%)	0 (0.00%)
Typhlopidae	*Anilios proximus*	0 (0.00%)	0 (0.00%)	1 (0.06%)	2 (0.13%)
	*Anilios nigrescens*	1 (0.06%)	0 (0.00%)	1 (0.06%)	0 (0.00%)
Pythonidae	*Morelia spilota* ssp. *metcalfei*	1 (0.06%)	0 (0.00%)	0 (0.00%)	0 (0.00%)
Elapidae	*Suta dwyeri*	9 (0.56%)	6 (0.38%)	3 (0.19%)	18 (1.14%)
	*Pseudonaja textilis*	12 (0.75%)	1 (0.06%)	4 (0.25%)	6 (0.38%)
	*Pseudechis porphyriacus*	3 (0.19%)	0 (0.00%)	0 (0.00%)	0 (0.00%)
Any species		760 (47.56%)	217 (13.73%)	141 (8.92%)	407 (25.74%)
**Number of surveys**		**1598**	**1581**	**1581**	**1581**

We detected the vast majority of individual reptile species only rarely. Of the 26 reptile taxa recorded between 2002 and 2020. The five species we most commonly detected were *M*. *boulengeri*, *C*. *marmoratus*, *C*. *spaldingi*, *C*. *tetradactyla*, and *C*. *pannosus* (Tables 1 and 2).

**Table 2:** Summary of detections of reptiles by year (Nomenclature follows Wilson and Swan 2020 [44]).

	Year	2002	2003	2005	2008	2011	2013	2016	2018	2020	Total
	**Number of surveys**	640	640	688	872	796	764	820	458	663	6341
**Family**	**Species**										
Agamidae	*Pogona barbata*	0	2	0	3	10	1	2	1	1	20
Carphodactylidae	*Underwoodisaurus milii*	0	0	1	1	1	1	1	0	0	5
Diplodactylidae	*Diplodactylus vittatus*	0	7	3	15	5	10	10	6	10	66
	*Strophurus intermedius*	0	0	1	0	3	0	1	0	0	5
Gekkonidae	*Christinus marmoratus*	44	43	31	41	16	32	38	20	16	281
Pygopodidae	*Delma inornata*	8	3	7	29	3	4	3	1	3	61
	*Aprasia parapulchella*	0	0	0	1	1	1	1	0	0	4
Scincidae	*Morethia boulengeri*	32	90	74	375	160	307	229	221	133	1621
	*Cryptoblepharus pannosus*	17	12	46	232	145	219	80	16	78	845
	*Carlia tetradactyla*	28	51	17	62	7	42	26	26	9	268
	*Ctenotus spaldingi*	3	3	22	22	44	40	15	7	7	163
	*Hemiergis talbingoensis*	7	17	13	15	9	8	23	14	29	135
	*Egernia striolata*	3	2	21	26	12	31	5	3	9	112
	*Lerista timida*	0	0	0	14	5	7	2	0	0	28
	*Lerista bougainvillii*	0	1	1	3	4	4	0	1	3	17
	*Tiliqua scincoides* ssp. *scincoides*	0	1	3	3	1	1	3	1	1	14
	*Menetia greyii*	2	2	0	5	0	2	0	0	1	12
	*Lampropholis delicata*	0	0	0	0	5	3	0	0	0	8
	*Lampropholis guichenoti*	2	0	0	1	0	0	0	2	1	6
Varanidae	*Varanus varius*	2	0	1	2	1	3	4	1	1	15
Typhlopidae	*Anilios proximus*	0	0	0	0	0	1	3	0	0	4
	*Anilios nigrescens*	1	0	0	0	0	0	0	0	1	2
Pythonidae	*Morelia spilota* ssp. *metcalfei*	0	0	1	0	0	0	0	0	0	1
Elapidae	*Suta dwyeri*	0	3	0	0	5	16	7	1	7	39
	*Pseudonaja textilis*	1	0	3	4	3	7	5	0	1	24
	*Pseudechis porphyriacus*	0	0	0	0	2	1	0	0	0	3
** **	**Totals**	150	237	245	854	442	741	458	321	311	3759

### Probability of absence and conditional and unconditional species richness and abundance

**Fig 2:**
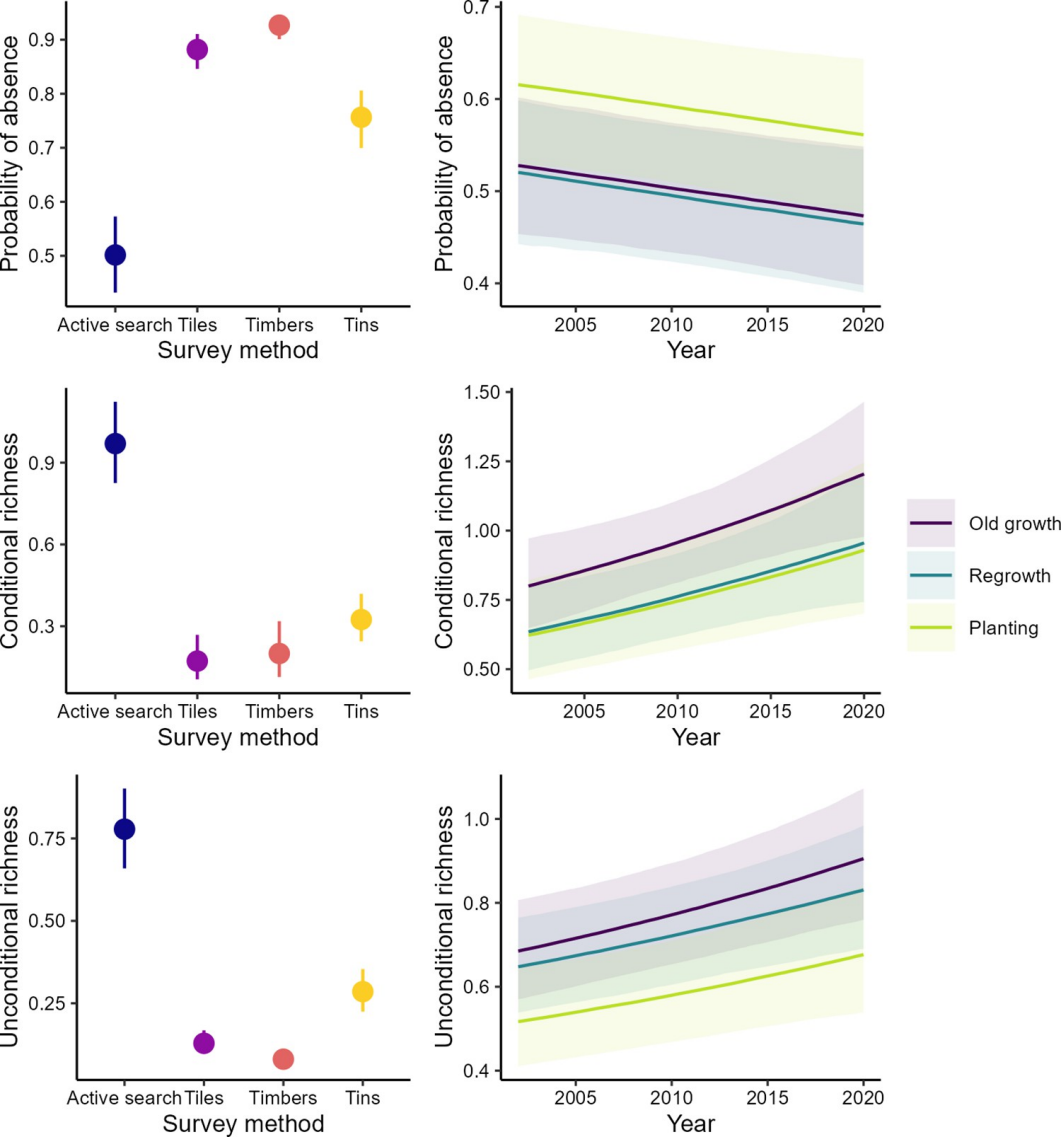
Relationships between the probability of absence, and conditional and unconditional species richness of reptiles in relation to search method, time, and growth type. We derived predictions from the best-fit model (Table 3).

**Fig 3:**
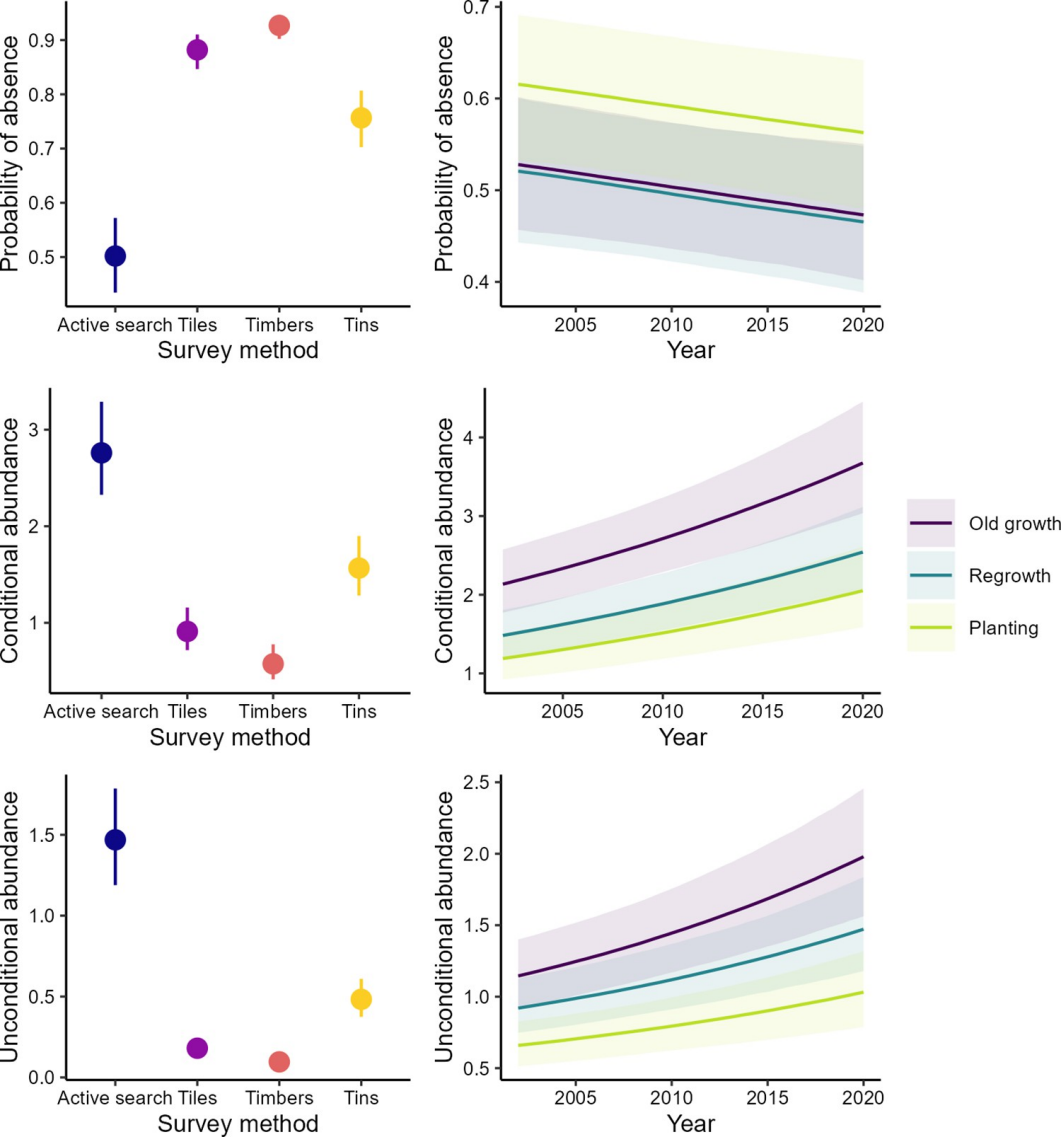
Relationships between the probability of absence, conditional abundance, and unconditional abundance of reptiles in relation to search method, time, and the interaction of time and growth type. We derived predictions from the best-fit model (Table 3).

The best fitting model for species richness and abundance contained no evidence of an interaction between time and growth type (Figs 2 and 3). That is, species richness and abundance increased over time in a similar way in all treatments as demonstrated by the probabilities of absence and conditional and unconditional species richness and abundance. Again, for both species richness and abundance, the probabilities of absence were lower when active searches were employed in surveys compared to when artificial arrays (i.e., tin, tiles or timber/wooden railway sleepers) were inspected. Conditional and unconditional richness and abundance all showed higher values for active searches than for the artificial substrates. That is, that the number of species and number of individuals observed during active searches was much greater than those observed using the substrates. Further to these effects, reptiles were more likely to be observed and exhibited greater richness and abundance in old growth woodland and regrowth woodland than in replantings (Figs 2 and 3).

**Table 3:** Model comparisons between the two models testing the linear and interactive effects of time and growth type. Bolded lines indicate best-fit model, as determined by lowest LOOIC score [41, 42].

Response variable	Model	LOOIC score
Species richness	**Time + growth type**	**7796.80**
	Time x growth type	7799.01
Abundance	**Time + growth type**	**10272.24**
	Time x growth type	10279.02
*Morethia boulengeri*	**Time + growth type**	**3774.76**
	Time x growth type	3776.62
*Christinus marmoratus*	**Time + growth type**	**1591.06**
	Time x growth type	1591.52
*Cryptoblepharus pannosus*	**Time + growth type**	**1376.42**
	Time x growth type	1378.90
*Carlia tetradactyla*	**Time + growth type**	**1587.96**
	Time x growth type	1590.70
*Ctenotus spaldingi*	**Time + growth type**	**674.50**
	Time x growth type	679.11

### Individual species responses

**Fig 4:**
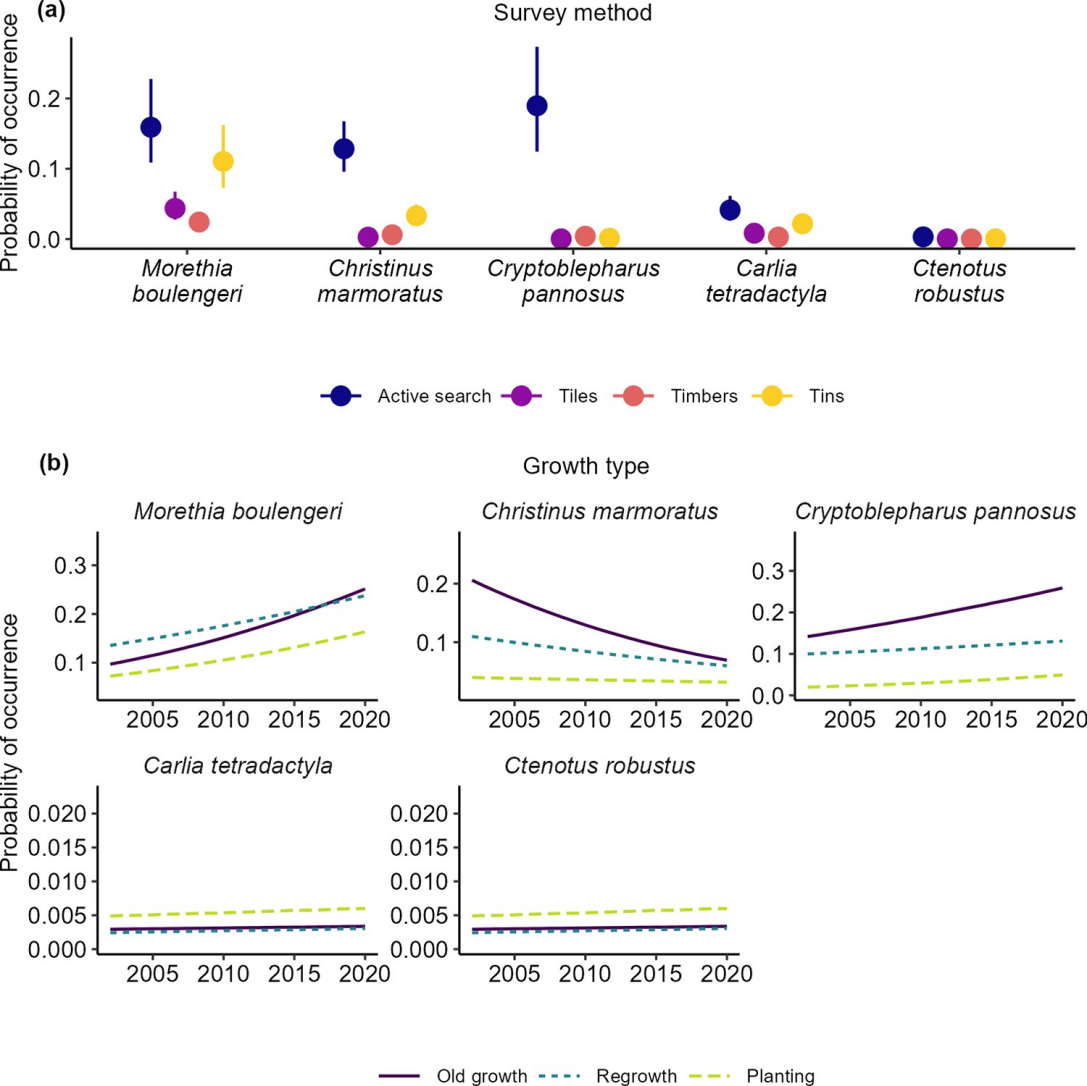
Individual species response predictions in response to: (a) different survey methods, and (b) growth types and over time. Predictions are based on the best-fit models in Table 3.

We found no evidence of an interaction between growth type and time for any of the individual species modelled (Table 2). There was decrease in detections between 2002 and 2020 for *C*. *marmoratus*, but a temporal increase in detections in *M*. *boulengeri* and *C*. *pannosus*. There was no temporal change in detections in *C*. *spaldingi* and *C*. *tetradactyla* (Fig 4).

**Fig 5:**
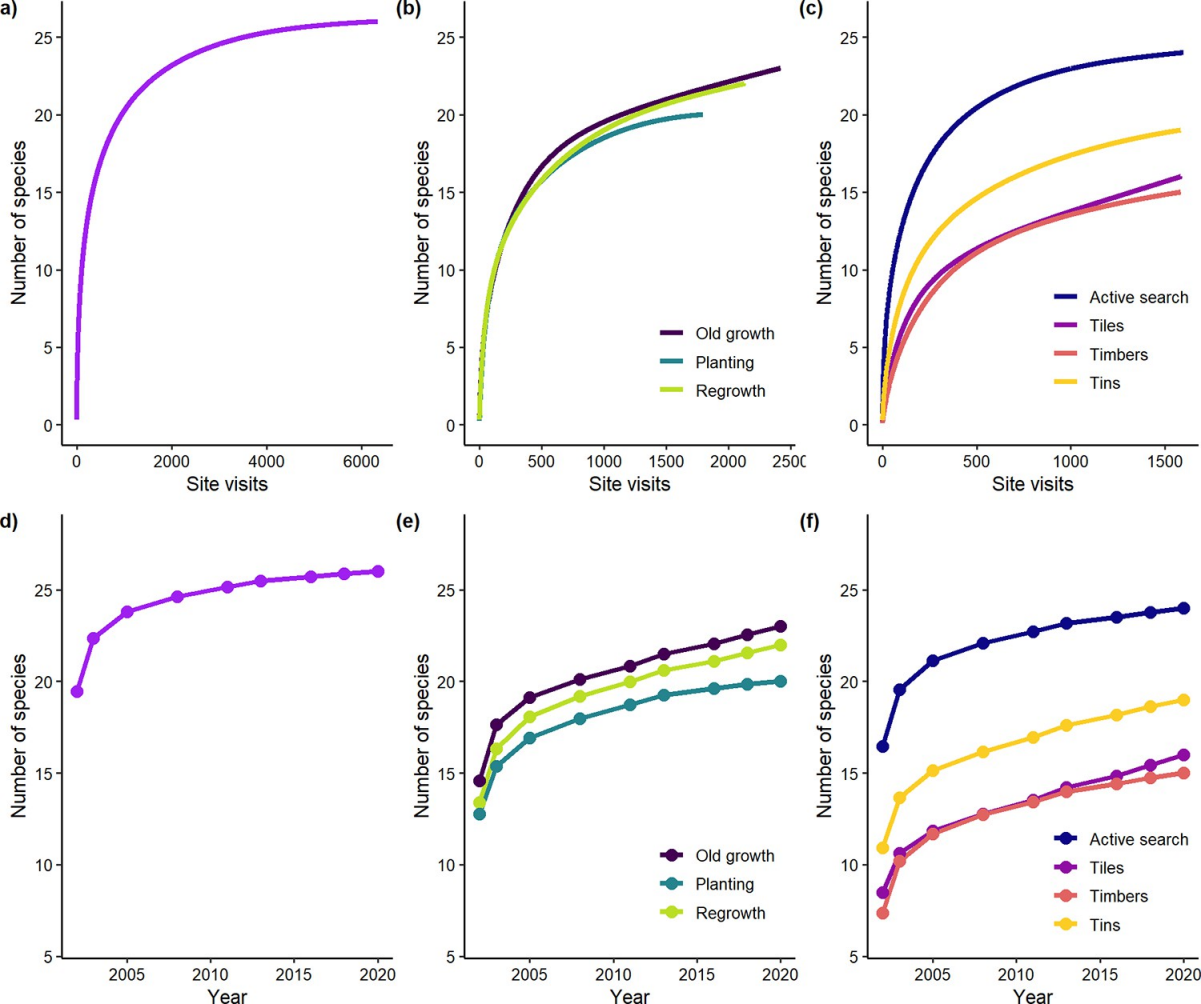
Species accumulation curves with increased site visits (a) overall, (b) in response to growth type, and (c) survey method. Species accumulation curves over the time of the study (d) overall, (e) according to growth type, and (f) survey method.

Our analyses revealed growth type effects for all five species that we analysed (Fig 5). *M*. *boulengeri*, *C*. *marmoratus*, *C*. *pannosus* were all least likely to be detected in replantings whereas *C*. *tetradactyla* and *C*. *spaldingi* were more likely to be recorded in replantings (Fig 4).

Finally, we found strong evidence of a survey method effect, with all species more likely to be detected by active searches than other survey methods (Fig 4).

### Species accumulation curves

Our species accumulation analysis revealed the accumulated total number of reptile species detected increased in response to: **(1)** the length of time surveyed, **(2)** survey effort over time in all broad vegetation types, but especially in old growth and regrowth woodland, and **(3)** survey effort over time under different survey methods (Fig 5).

## Discussion

Understanding patterns of temporal change in biota and quantifying the factors influencing species distribution and abundance are fundamental components of the disciplines of ecology [45, 46] and conservation biology [3, 47]. Such data, especially on patterns of temporal change in occurrence, are scant for many groups of biota in Australia, including terrestrial reptiles [6]. We sought to quantify temporal changes in reptile biota in the temperate woodlands of the SWS bioregion of southern New South Wales. The key findings of this study were that: **(1)** There were relatively few reptile detections, with the majority of surveys recording no species, especially using tin, timber, and tiles substrates. **(2)** There were no differences in the trajectories of reptile detections over time among broad vegetation types. **(3)** At the individual species level, there was a trend for increasing detections over time for some species, but declines in others. Some species were more likely to be recorded in replantings whereas others were less likely to be detected in replantings. And, **(4)** The accumulated number of species of reptiles increased with time and survey effort, especially in old-growth woodland and regrowth woodland. We discuss these key results in the remainder of this paper and conclude with some commentary on the broad management implications of the results reported here.

### Patterns of temporal change and growth type effects

Our analyses suggested there were “winners and losers” in woodlands with evidence of increases in detections for two skink species, a decline in one gecko species, and no temporal change in the remaining two skink species. This result is broadly consistent with findings across multiple groups globally, which suggest that some species in a given landscape, ecosystem or biome increase over time whereas others decline [e.g. 48]. The two species of skinks exhibiting a stable temporal trend were associated with replantings; such areas typically have more grass cover and are subject to less grazing pressure relative to the other broad vegetation types in our study, and these characteristics may favour population stability [49]. Interestingly, there was a decline in the gecko species, *C*. *marmoratus* which is arboreal and often associated with large healthy trees. In contrast, another arboreal reptile, *C*. *pannosus* that prefers dead and dieback-affected trees increased along with another species (*M*. *boulengeri)* that is adapted to open and relatively dry habitats. However, despite extensive field survey effort, very few reptile species were detected sufficiently frequently to enable detailed analyses of relationships between temporal and spatial occurrence and life history attributes like behaviour, habitat preferences and food resources (that can otherwise be tractable through approaches like fourth corner analysis; [e.g. see 50]). On this basis, it is currently not possible to determine the key factors explaining differences between positive and negative temporal patterns in occurrence. Our analysis revealed evidence of a temporal increase in composite measures of conditional (given presence) and unconditional species richness and abundance in all broad vegetation types (Figs 2 and 3). An interesting outcome was that the rate of increase was similar in replantings as in regrowth woodland and old growth woodland. This highlights the value of restored (replanted) areas in contributing to improved woodland reptile biodiversity [see 31, 51]. Our results also demonstrate that management interventions like restoration replantings have value in contributing to biodiversity conservation–as shown in earlier studies on other groups such as woodland birds [see 34, 52]. An important caveat is that how replantings are managed can have a major impact on reptile occurrence; when they are grazed by domestic livestock their value as habitat for reptiles can be eroded significantly [49].

Beyond the positive effects of replantings for reptiles, our analyses indicated that for some individual species such as *C*. *marmoratus*, detections were greater in old-growth woodland than regrowth and replantings. This is likely to be closely linked with the habitat requirements of those species. For example, *C*. *marmoratus* is arboreal and often found on large trees with flaking bark [12, 20]–attributes more likely to occur in old-growth woodland relative to other broad structural vegetation types [28]. More detections of individual species would be required to allow construction of complex statistical models that included measures of detailed vegetation structure and analyses beyond ours of broad vegetation structure types (i.e., old growth woodland, regrowth woodland, and replantings).

### Species accumulation curves

We recorded 26 species of reptiles in the nine survey periods conducted between 2002 and 2020. Analyses of species accumulation curves showed that new species continued to be recorded over time. We are aware that this number would likely continue increasing with further survey effort and time as there are ~ 15 other species which occur in the broader region, but which have not been detected in our surveys to date [see 12]. However, our data show that many surveys (~ 2000 site x year surveys) were required to exceed 20 species reptiles being detected (Fig 5). This large effort emphasises the general rarity of detections of reptiles in the surveys we conducted and the extensive amount of effort expended to gather data on this group in temperate agricultural landscapes. Our results also suggest that some species of reptiles may have declined substantially or become locally extinct on the farmlands where we completed repeated surveys. Notable absences include several fossorial or lizard-eating (saurophagous) Elapids and Pygopodids, and small Agamids.

Increases in the number of species recorded over time were less marked in replantings, possibly because such areas typically support a less diverse array of habitats than those which characterise old growth and regrowth woodland. Species accumulation curves underscore the difficulties in working with reptiles in temperate woodland environments and the amount of survey effort needed to gain an understanding of what species occupy a given area [53, 54].

### Paucity of reptile detections

A particular feature of our long-term study was the paucity of detections of reptiles in the vast majority (~75%) of surveys. This figure is heavily influenced by the relatively low number of detections in the tile substrate (13.7% detections) and timber substrate (8.9%). However, even using active searches, which is the most effective detection method [see 37], the detection rate was still less than half of the surveys (47.6%). The maximum number of species detected at a site in any given survey was five (of an estimated 41+ species in woodlands in the region = 12.2%). This was proportionally similar to, for example, to maximum number of bird species detected at the same sites we surveyed for reptiles, 31 bird species per site per survey (or 17.5% of the 177 bird species recorded in the region) [up to 25 bird species per site per survey; 25, 29]. Notably, only five individual species of reptiles were sufficiently common to enable subsequent statistical analyses of field data—a common problem found in many reptile studies, and not unique to Australian agro-ecosystems [e.g. see 55, 56]. This result may be an outcome of the general rarity and filtering of reptiles in agriculture-dominated landscapes where there has been extensive modification of the vegetation and/or from the low detectability of reptiles in the types of surveys we completed. However, we acknowledge that despite the use of a range of well-established field survey methods (see below), some species which are relatively common in agricultural landscapes, such as *Pseudonaja textilis* [57], were likely missed and probably somewhat more common than reflected in our datasets. In this case (and possibly other cases), specifically targeted, “fit-for-purpose” surveys using pitfall and funnel traps might yield greater numbers of detections than generated by our broadly based general survey protocols. Moreover, some habitat types which we did not explicitly survey in this study such as rocky outcrops can be hotspots for reptiles [19, 58]. Subsequently, surveys more targeted at such places would likely produce more detections than we have reported here.

### Survey method effects

Consistent with an earlier analysis of our reptile data, we found active searches resulted in the detection of more species and more individuals of a particular species than other survey methods [see 37]. This result was expected as active searches are conducted for longer and over a greater area than other kinds of surveys, involve inspection of more kinds of habitats than any one kind of artificial substrate type, and allow the inclusion of basking and actively moving animals [37]. However, our data indicated that some rarer species were more likely to be detected using particular kinds of substrates. For example, *Suta dwyeri* was associated with tin, and *Diplodactylus vittatus* and *Delma inornata* with tiles (Table 1).

## Conclusions

We report the results of extensive surveys for reptiles in the temperate woodlands of south-eastern Australia that spanned 218 sites in a 1.5 million ha area across an 18-year period (between 2002 and 2020). Despite extensive sampling effort, the majority of surveys produced no detections of animals, an outcome possibly associated with the extent of human disturbance in temperate woodland environments and prior filtering of the reptile assemblage. Significant survey effort over a prolonged period of time was needed to record detections of more than 20 species of reptiles in the study area. Our analyses showed a temporal increase in species richness and abundance over time. Patterns of temporal change were similar in all three broad vegetation structural types we surveyed. Our data highlights the importance of all three broad structural types of woodland cover for reptiles in agricultural landscapes.
